# Wiedemann–Steiner Syndrome (WSS): A Neonatal Case Report Expanding the Phenotypic Spectrum of a Previously Reported Missense Variant

**DOI:** 10.3390/ijms27104163

**Published:** 2026-05-07

**Authors:** Myrto Poulou, Thessalia Kamilari, Eirini Nikaina, Eleftheria Dikoglou-Tzanetatou, Christina Kanaka-Gantenbein, Aggeliki Kolialexi, Tania Siahanidou

**Affiliations:** 1 Department of Genetics, Institute of Child Health, 11527 Athens, Greece; e.ditza@ich.gr (E.D.-T.); a.kolialexi@ich.gr (A.K.); 2 1st Department of Pediatrics, Medical School, National and Kapodistrian University of Athens, “Agia Sophia” Children’s Hospital, 11527 Athens, Greece; kamilarithessalia@gmail.com (T.K.); enikaina@gmail.com (E.N.); chriskan@med.uoa.gr (C.K.-G.); siahan@med.uoa.gr (T.S.)

**Keywords:** Wiedemann–Steiner Syndrome (WSS), *KMT2A*, de novo missense variant, interstitial lung disease, hypertrophic pyloric stenosis

## Abstract

We present a neonatal case of Wiedemann–Steiner syndrome (WSS) with a de novo, previously reported *KMT2A* missense variant (c.3464G>A; p.Cys1155Tyr; NM_001197104.2), and provide a focused literature review of this specific variant. WSS (OMIM#605130) is a rare neurodevelopmental disorder caused by heterozygous variants in the *KMT2A* gene, which encodes a histone H3 lysine K4 (H3K4) methyltransferase involved in transcriptional regulation. Clinically, the syndrome is characterized by developmental delay, distinctive facial features, short stature, hypertrichosis, and neurological manifestations such as hypotonia and seizures. In this single-patient report, we describe additional clinical findings, including interstitial lung disease and hypertrophic pyloric stenosis requiring surgical intervention. These may represent rare manifestations of WSS that require confirmation in further reports before a formal expansion of the phenotypic spectrum can be established. Most pathogenic *KMT2A* variants arise de novo and are typically nonsense or frameshift; however, missense variants have also been reported and may have complex functional consequences. Haploinsufficiency is considered the primary pathogenic mechanism, leading to the disruption of chromatin modification and transcriptional regulation. While emerging genotype–phenotype correlations are being identified, considerable variability remains. Given the single-patient nature of this study, these observations should be considered hypothesis-generating and require confirmation in additional cases.

## 1. Introduction

Wiedemann–Steiner syndrome (WSS; OMIM #605130) is a rare genetic disorder resulting from heterozygous variants (autosomal dominant inheritance) in the *KMT2A* gene, which encodes a histone H3 lysine 4 (H3K4) methyltransferase, essential for transcriptional regulation [1,2]. Through H3K4 methylation, *KMT2A* promotes an open chromatin configuration that facilitates gene expression during cellular differentiation and development [3,4,5].

Accordingly, WSS is classified among chromatinopathies, a group of disorders resulting from defects in epigenetic machinery [6]. Since the first clinical description of this syndrome in 1989, advances in genomic technologies, particularly whole-exome sequencing (WES), have significantly improved the diagnostic recognition [2,5,7,8].

Clinically, WSS is characterized by a variable but recognizable phenotype that typically includes developmental delay, intellectual disability, distinctive craniofacial features, short stature, and hypertrichosis particularly involving the elbows. Neurological involvement is common and may include hypotonia and seizures [3,9,10]. Additional manifestations may include feeding difficulties, skeletal anomalies, and multisystem congenital abnormalities [11]. Expanding evidence also highlights endocrine and immunological involvement, further broadening the phenotypic spectrum [12,13,14].

Most pathogenic *KMT2A* variants arise de novo and include truncating and missense changes. Missense variants frequently cluster within functionally critical domains, particularly the CXXC zinc-finger domain, which mediates binding to unmethylated CpG regions and is essential for gene regulatory activity [15,16,17]. The disruption of this domain has been associated with significant alterations in chromatin regulation and may contribute to the genotype–phenotype variability observed in WSS [17].

Despite increasing recognition of WSS, neonatal presentations and early systemic manifestations remain underreported. Further well-characterized cases are therefore important for refining the clinical spectrum and improving early diagnosis. Here, we report a neonatal case of WSS caused by a de novo *KMT2A* missense variant (c.3464G>A; p.Cys1155Tyr) affecting the CXXC domain. In addition to typical features, the patient presented with interstitial lung disease and hypertrophic pyloric stenosis requiring surgical intervention. These findings may represent rare or previously underrecognized manifestations and warrant further investigation in additional cases.

## 2. Detailed Case Description

A 33-day-old full-term female infant, delivered vaginally to consanguineous parents, was referred to our Neonatal Intensive Care Unit (NICU) for further evaluation of failure to thrive, vomiting, and refractory hypoxemia.

At birth, the infant’s anthropometric measurements were as follows: weight, 2250 g; length, 46 cm; and head circumference, 32 cm, all of which corresponded to the third percentile. The amniotic fluid was meconium-stained, and the patient presented with respiratory distress at birth, requiring oxygen supplementation via a hood and free flow. Meconium aspiration syndrome (MAS) was suspected; however, chest X-ray findings were not supportive, as neither bilateral lung opacities nor significant hyperinflation were observed. The patient was treated with intravenous ampicillin and gentamicin for suspected early-onset neonatal sepsis which was subsequently excluded. On the 7th day of life, respiratory deterioration was noted, requiring oxygen supplementation via a high-flow nasal cannula (HFNC). A repeat septic screen was negative and a real-time Polymerase Chain Reaction (PCR) testing for respiratory viruses did not identify any pathogens.

**Figure 1:**
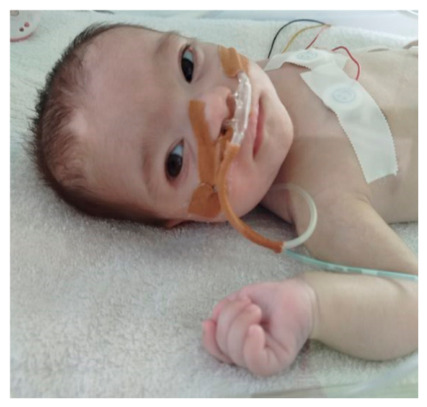
Clinical characteristics of the patient.

Upon admission to our unit, hypotonia, hypertelorism, high-arched palate, low-set ears, short philtrum, cubitus hypertrichosis, and widely spaced nipples were observed on clinical examination (Figure 1).

**Figure 2:**
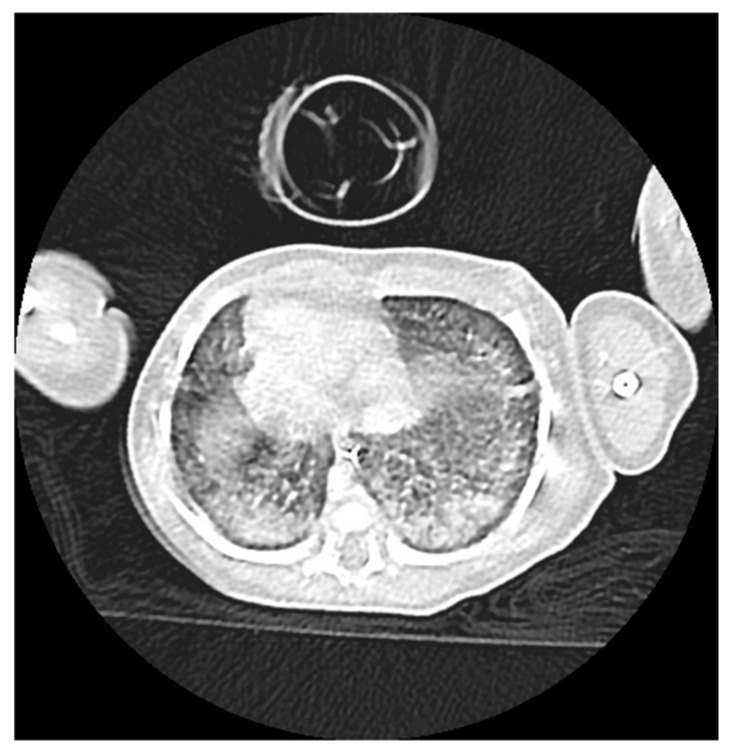
Chest computed tomography (CT) demonstrating bilateral diffuse ground-glass opacities with relative subpleural sparing.

**Figure 3:**
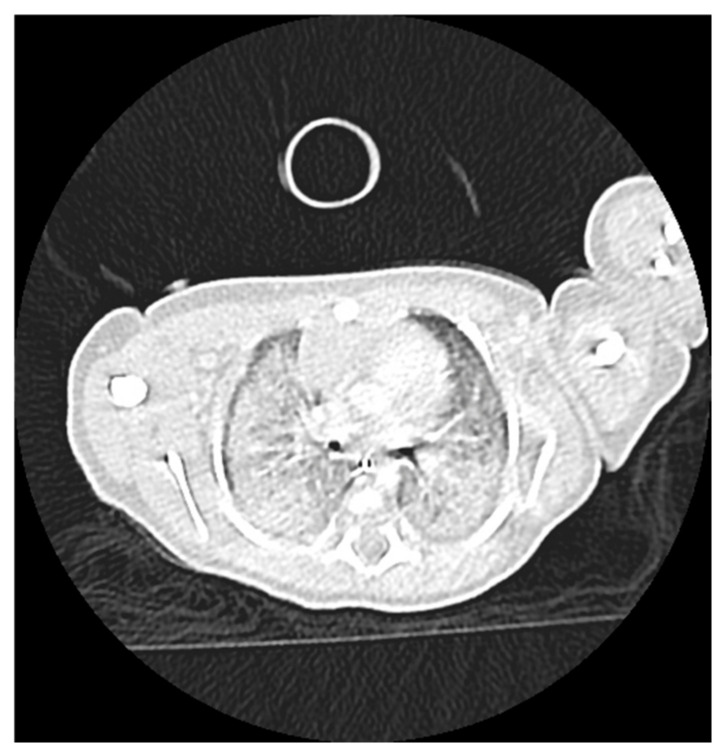
Chest computed tomography (CT) demonstrating bilateral diffuse ground-glass opacities with relative subpleural sparing and fibro-atelectatic changes in the right lower lobe.

Chest X-ray revealed pulmonary opacities in both lungs, whereas a chest CT demonstrated bilateral diffuse infiltrates with ground-glass opacities sparing the subpleural regions, and fibro-atelectatic changes in the anterior basal segment of the right lower lobe. The trachea and main bronchi were patent and no pleural or pericardial effusion was detected (Figure 2 and Figure 3). These findings favored a diagnosis of interstitial lung disease (ILD). The arterial blood gas analyses were consistent with ILD, demonstrating decreased pO2, in the absence of hypercapnia or acid-base disturbance. Brain–lung–thyroid (BLT) syndrome was excluded as thyroid function tests were within normal limits. Serum α1-antitrypsin levels (1.2 g/L) were also normal, whereas bronchopulmonary dysplasia (BPD) was considered unlikely given that it primarily affects preterm infants.

**Figure 4:**
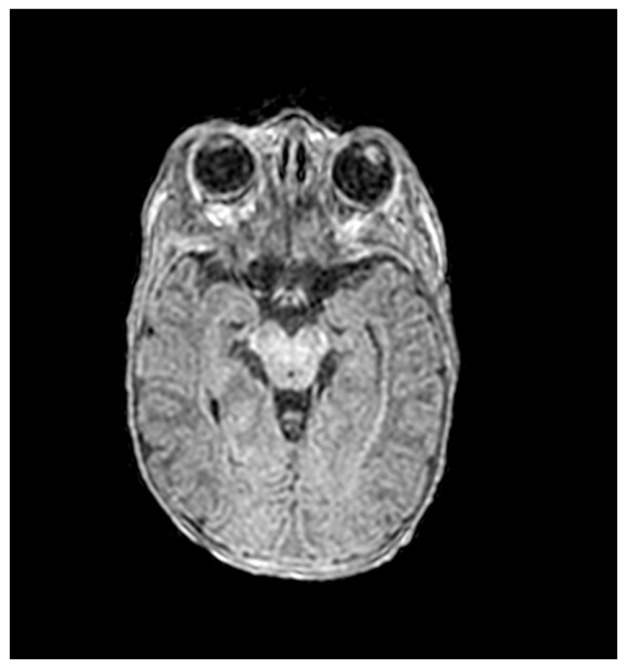
Brain magnetic resonance imaging (MRI) showing no pathological findings.

**Figure 5:**
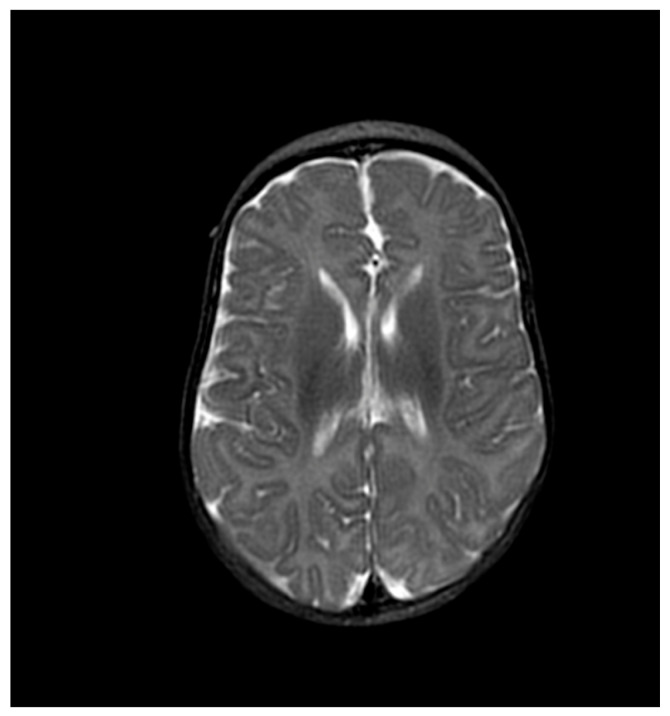
Brain magnetic resonance imaging (MRI) showing no pathological findings.

Echocardiography revealed a small patent ductus arteriosus (PDA) measuring 0.6 mm, with continuous left-to-right flow and a peak systolic gradient of 28.5 mmHg. Ophthalmological examination, auditory brainstem response (ABR), and brain magnetic resonance imaging (MRI) were all normal (Figure 4 and Figure 5).

**Figure 6:**
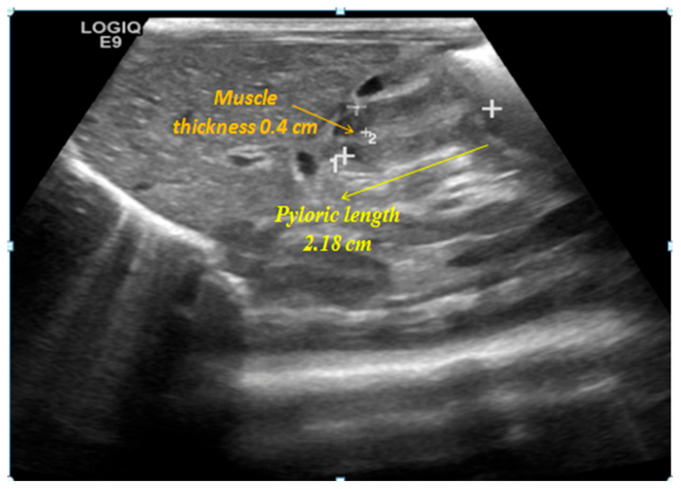
Abdominal ultrasonography demonstrating hypertrophic pyloric stenosis.

Since birth, the patient exhibited absent sucking and gag reflexes, rendering breast or bottle feeding impossible. Repeated attempts at bottle feeding were unsuccessful. The patient was able to swallow only minimal amounts of milk via bottle feeding under caregiver-applied pressure, whereas the remainder was expelled due to uncoordinated tongue movements. Exclusive nasogastric tube feeding was well-tolerated and enteral nutrition was fully established by the end of the first week of life. Failure to thrive was noted, in the absence of other gastrointestinal symptoms. The patient remained clinically stable regarding enteral intake until the 27th day of life, when there was a sudden onset of persistent regurgitation and projectile vomiting, without any preceding signs of feeding intolerance or irritability. An abdominal ultrasonography was performed revealing findings consistent with hypertrophic pyloric stenosis, with a pyloric muscle thickness of 0.4 cm and a pyloric length of 2.18 cm (Figure 6). The patient underwent surgical management with pyloromyotomy. Postoperatively, vomiting episodes ceased; nevertheless, feeding issues and suboptimal weight gain persisted. A hypercaloric formula was initiated via nasogastric tube, resulting in improved weight gain.

**Figure 7:**
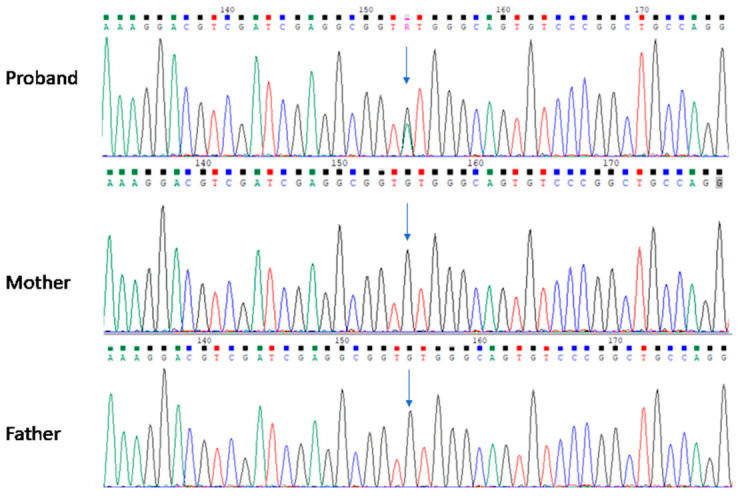
Sanger sequencing traces of exon 5 of *KMT2A* (NM_001197104.2), revealing the de novo nature of the variant p.Cys1155Tyr (arrow; c.3464G>A).

Given the high clinical suspicion of an underlying genetic disorder and the known genetic basis of several causes of neonatal ILD, including surfactant dysfunction disorders, pulmonary alveolar proteinosis, primary ciliary dyskinesia, and acinar dysplasia, a genetic analysis was warranted [18]. WES was performed, which revealed a heterozygous germline mutation in exon 5 of the *KMT2A* gene, c.3464G>A (p.Cys1155Tyr), associated with Wiedemann–Steiner syndrome. To determine the inheritance pattern, parental genetic testing using Sanger sequencing (SeqStudio Genetic Analyzer—Thermo Scientific, Waltham, MA, USA) was conducted. Both parents tested negative, suggesting that the identified pathogenic variant in the patient arose de novo (Figure 7). According to ACMG/AMP guidelines [19], this variant is classified as pathogenic based on multiple lines of evidence, including its de novo occurrence (PS2), location within a critical and well-established functional domain (PM1), previous reports in affected individuals (PS4), and computational evidence supporting a deleterious effect (PP3). In addition to the identified *KMT2A* variant, the WES data were systematically analyzed for other clinically relevant variants, and no additional pathogenic or likely pathogenic variants were detected that could independently explain the observed phenotype, reducing the likelihood of a dual genetic diagnosis.

Following the diagnosis of WSS, immunological testing was performed as part of a broader diagnostic workup. Serum immunoglobulin levels were within the normal range for age. Mild lymphocytosis was noted (maximum 11,170 lymphocytes/μL), with a proportional increase across all major lymphocyte subsets on peripheral blood immunophenotyping, without evidence of immunodeficiency. Specifically, the proportion of activated T-lymphocytes was normal and the CD4^+^/CD8^+^ T-cell ratio was within normal limits. Notably, the percentage of T-lymphocytes expressing the TCRγδ receptor was normal, as was the proportion of double-negative T-lymphocytes, whereas HLA-DR expression within this population was preserved. Given that immunological abnormalities may present later on in patients with WSS, a follow-up immunological evaluation of our patient was scheduled.

At 3 months of age, oxygen supplementation was discontinued. A gastrostomy feeding tube was placed to address persistent oral feeding difficulties. The infant was discharged in a stable condition. Given the complexity of her presentation and the confirmed diagnosis of WSS, a multidisciplinary approach and follow-up surveillance were recommended to ensure optimal patient management.

## 3. Discussion

The *KMT2A* gene, located on chromosome 11q23, encodes a transcriptional coactivator that plays an essential role in regulating gene expression during early development and hematopoiesis [20]. Transcriptional activation is mediated by the addition of methyl groups to histone H3 lysine 4 (H3K4), a process that marks and opens active gene promoters [17,21].

*KMT2A* alterations are associated with two distinct clinical contexts:

(a) Leukemia: *KMT2A* rearrangements and partial tandem duplications (*KMT2A*-PTD) are hallmark mutations in high-risk pediatric, infant, and therapy-related AML/ALL. “Numerous genomic breakpoints within the *KMT2A* gene have been reported in young children and adults with hematologic disorders and are present in up to 10% of acute leukemias” [22].
(b) Developmental Syndrome: Heterozygous germline mutations in the *KMT2A* gene (functional loss of one copy) lead to WSS [3,5], a neurodevelopmental disorder characterized by developmental delay, distinctive facial features, short stature, hypertrichosis, and neurological features, including hypotonia and seizures [2]. Our case adds to the phenotypic spectrum of the syndrome, as it presented with characteristic interstitial lung disease and hypertrophic pyloric stenosis requiring surgical intervention.

Most pathogenic variants arise de novo, and are mainly nonsense and frameshift variants; however, missense variants, as in this case, with potentially complex effects have also been reported. Notably, missense variants cluster within the CXXC domain (amino acids 1147–1242), making it a recognized mutational hotspot [11,17,23]. As of 1 March 2026, the professional Human Gene Mutation Database (HGMD^®^) [24] (available online: https://my.qiagendigitalinsights.com/bbp/view/hgmd/pro/gene.php?gene=KMT2A accessed on 1 March 2026) lists 342 *KMT2A* variants associated with Wiedemann–Steiner syndrome, including 100 missense variants, of which 37 are located between amino acids 1147–1195.

**Figure 8:**
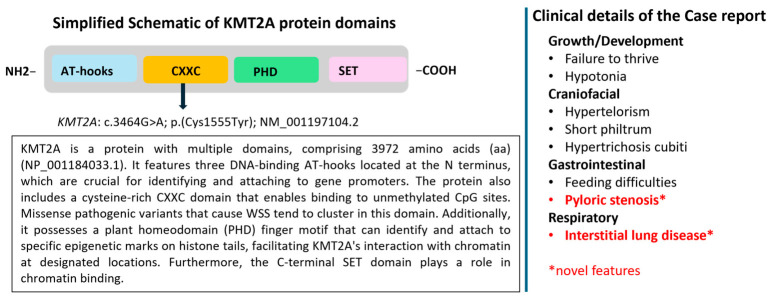
Simplified schematic representation of KMT2A protein domains, their functional roles, and the clinical features observed in the present case. Asterisks (*) indicate novel features reported in this study.

In WSS, mutations in the *KMT2A* gene disrupt its function as a histone methyltransferase; haploinsufficiency remains the principal pathogenic mechanism, leading to downstream disruption of chromatin modification [4,23,25]. The CXXC domain is frequently affected [8], suggesting that the loss of its ability to bind unmethylated CpG dinucleotides is a key component of the molecular pathogenesis of the syndrome (Figure 8). Other protein domains of *KMT2A* are also involved, as the entire protein plays a role in chromatin remodeling and gene regulation, which are essential for development [9,17,23,26].

Our proband carries a de novo heterozygous *KMT2A* c.3464G>A (p.Cys1155Tyr) variant, identified by whole-exome sequencing (WES) and confirmed by Sanger sequencing of the proband and parental samples. This variant affects the CXXC [23] zinc-finger DNA-binding domain, which is critical for CpG binding and the regulation of histone methylation. The disruption in this region has been consistently linked to WSS, and the specific variant has been independently reported in several cohorts and individual studies. Studies have shown that the CXXC domain is highly intolerant to variation. Patients carrying missense variants located in the CXXC domain (especially amino acids 1147–1195) often present with a “more severe neuro-phenotype” compared to those with truncating mutations [2,15,16,27]. Pathogenic missense variants within the CXXC domain exhibit a strong genotype–phenotype correlation, particularly when compared to other domains. Patients with these variants typically present with severe intellectual disability, feeding difficulties, and short stature, in contrast to those with missense variants in other domains or splice variants [2,5,13,15,16,28].

Table 1 provides a comprehensive overview of studies reporting the *KMT2A* c.3464G>A (p.Cys1155Tyr) variant. Comparison with previously reported cases carrying the same variant indicates that the core phenotype is largely consistent, particularly with respect to neurodevelopmental impairment, feeding difficulties, and characteristic dysmorphic features. Notably, pulmonary involvement and hypertrophic pyloric stenosis have not been previously reported in association with this variant, suggesting a potential expansion of the phenotypic spectrum. These findings may represent either rare manifestations or previously underrecognized features, highlighting the importance of detailed phenotyping and case aggregation. In previously reported cases, abnormal morphological characteristics and PDA were apparent from birth, whereas other features, including feeding difficulties, failure to thrive or hypotonia, became more evident during infancy. In contrast, our patient exhibited multisystem involvement already in the neonatal period. Intellectual disability, short stature and/or recurrent infections were reported later in infancy or during childhood.

The coexistence of interstitial lung disease and hypertrophic pyloric stenosis in this patient raises the possibility of broader systemic involvement in rare cases of *KMT2A* haploinsufficiency, although a coincidental association or contribution of perinatal factors cannot be excluded. *KMT2A*, as a member of the COMPASS family of H3K4 methyltransferases, is critical for the activation of developmental gene expression programs through chromatin modification [29,30]. Haploinsufficiency may impair the transcriptional regulation of genes involved in tissue-specific differentiation and morphogenesis. During embryogenesis, lung development depends on tightly regulated epithelial–mesenchymal interactions, branching morphogenesis and alveolar differentiation, processes known to be under epigenetic control [31,32]. The Disruption of chromatin regulation and H3K4 methylation may therefore affect pulmonary epithelial maturation and structural organization, potentially contributing to interstitial lung pathology [33]. Similarly, pyloric and broader gastrointestinal development require coordinated smooth muscle differentiation and tightly regulated signaling pathways, including Hedgehog, Wnt, and Notch pathways, which are essential for gut patterning and organogenesis [34,35]. Epigenetic dysregulation of these pathways could interfere with gastrointestinal patterning and muscular development, providing a plausible mechanistic explanation for hypertrophic pyloric stenosis. Furthermore, as the identified variant affects the CXXC domain, which is essential for CpG binding and genomic targeting, it may result in locus-specific transcriptional dysregulation of developmental genes relevant to both pulmonary and gastrointestinal systems. Although these mechanisms remain speculative, they are consistent with the expanding phenotypic spectrum observed in chromatinopathies and highlight the potential for broader systemic involvement in WSS [6].

Beyond structural abnormalities detectable on conventional imaging, chromatin regulators such as *KMT2A* play a central role in neurodevelopmental processes, including neuronal differentiation, synaptic plasticity, and large-scale brain network organization [36,37,38]. This may reflect network-level neurodevelopmental alterations that are not detectable on structural imaging, as has been proposed in other neurodevelopmental disorders [6,36]. Emerging evidence suggests that such functional and connectivity changes may precede or occur independently of structural findings [39].

In addition to transcriptional regulation, emerging evidence indicates that epigenetic modifications, such as H3K4 methylation, are closely linked to co-transcriptional RNA processing, including alternative splicing [40]. This interaction is particularly relevant in neuronal tissues, where alternative splicing contributes to proteomic diversity and functional specialization. The disruption of these processes has been increasingly implicated in neurodevelopmental and neurodegenerative disorders [41]. Recent studies suggest that chromatin regulators and the spliceosomal machinery are functionally interconnected, with histone modifications influencing exon selection and transcript diversity [42]. In this context, *KMT2A* dysfunction may not only alter gene expression at the transcriptional level but also indirectly affect RNA processing networks, thereby contributing to the complex neurological phenotype observed in WSS [43].

Nevertheless, alternative explanations for the observed respiratory and gastrointestinal manifestations should be considered. The patient’s low birth weight and early respiratory distress may independently predispose to neonatal pulmonary complications, including interstitial-like radiographic changes or delayed lung maturation [18]. Likewise, feeding difficulties and vomiting are relatively common in neonates with hypotonia or prematurity-related functional immaturity of the gastrointestinal tract [44]. Although hypertrophic pyloric stenosis is a distinct structural condition, its coexistence with feeding intolerance in early infancy may complicate clinical interpretation [45]. Therefore, while a unifying genetic mechanism is plausible, the contribution of perinatal and non-genetic factors cannot be excluded.

Importantly, this report represents a single-patient observation, and definitive genotype–phenotype correlations cannot be established. The association of p.Cys1155Tyr with pulmonary and gastrointestinal involvement should therefore be considered hypothesis-generating and requires validation in larger cohorts.

**Table 1:** Comparison of previously reported cases with *KMT2A* NM_001197104.2:c.3464G>A (p.Cys1155Tyr) and the present case.

Source (Citation)	Case ID/Cohort	Inheritance	Age/Sex	Key Phenotypes Reported	Evidence/Notes
Present case (this report)	Proband	De novo	Neonate (female)	failure to thrive, hypotonia, dysmorphic features, feeding difficulties, patent ductus arteriosus, hypertrichosis cubiti, **hypertrophic pyloric stenosis, interstitial lung disease**	WES confirmed; detailed clinical phenotype
Foroutan A et al. [26] PMID: 35163737	WDSTS_EPIC Pt.7	Not specified (likely de novo)	Male, 3 years	included in methylation episignature cohort; phenotype details limited	Variant validated within cohort
Kaur A et al. [27] PMID: 38567171	WDST	De novo	Female, 11 months	small palpebral fissures, thin upper lip, hypertrichosis cubiti, puffy hands, hypotonia, failure to thrive, global developmental delay	WES confirmed
Baer S et al. [25], PMID: 29574747.	WDST, pt.25		Male, 5 years	hypertelorism, small palpebral fissures, downslanted palpebral fissures, thick eyebrows, long eyelashes, thin upper lip, advance bone age (+2 years), rib anomalies (11 pairs), tapering finger, sacral dimple, hypertrichosis of the back, hypertrichosis of lower limbs, hypotonia neonatal and persistent, developmental delay, severe intellectual disability, seizures (absences), dysgenesis of corpus callosum, bilateral ptosis, strabismus, astigmatism, lachrymal stenosis, left pyelectasia, posterior urethral valve, constipation, frequent infections, central apneas	WES
Bramswig NC et al. [46]; PMID: 25724810.	K2431–WDST	De novo	Male, 22 months	intellectual disability, hypotonia, right retinal atrophy, frequent infections, feeding problems, coarse face, low frontal hairline, thick eyebrows, long eyelashes, flat nasal bridge, broad nose, upturned nasal tip, large mouth, thin upper vermillion, thick lower vermillion, macroglossia, long philtrum, small, protruding ears, aplasia/hypoplasia of distal phalanges of the 5th finger, prominent interphalangeal joints, prominent distal phalanges, cryptorchidism, patent ductus arteriosus, mitral valve prolapse, body hirsutism, sparse scalp hair, fasciculation of tongue	WES Trio-analysis/the patient presented aspiration pneumonia at the age of 3 months and recurrent pulmonary infections—died of sepsis at the age of 3 years.
Li et al. [16] PMID: 30305169	Chinese cohort	Not specified	Not detailed	cohort phenotypes: developmental delay, hypertrichosis, short stature; phenotype details limited for specific variant	Variant listed in supplemental tables

## 4. Conclusions

This report describes a neonatal presentation of Wiedemann–Steiner syndrome associated with the recurrent *KMT2A* c.3464G>A (p.Cys1155Tyr) variant and expands the clinical spectrum of this condition. In addition to the established core phenotype, the presence of interstitial lung disease and hypertrophic pyloric stenosis in this case highlights the importance of considering broader multisystem involvement in some patients with WSS, although whether these findings are causally related remains uncertain.

While these observations raise the possibility of an expanded genotype–phenotype correlation for variants affecting the CXXC domain, current evidence remains insufficient to establish a causal relationship. Given that this is a single-patient observation, no definitive genotype–phenotype correlation can be established, and these associations should be considered hypothesis-generating, pending confirmation in additional cases.

This case underscores the value of comprehensive genomic testing, such as whole-exome sequencing, in the diagnostic evaluation of complex neonatal presentations, particularly in the context of multisystem involvement [47]. Systematic phenotyping and aggregation of similar cases will be essential to refine the clinical spectrum of *KMT2A*-related disorders and to improve early recognition, prognostic assessment, and patient management.

## Data Availability

The original contributions presented in this study are included in the article. Further inquiries can be directed to the corresponding author.

## Abbreviations

The following abbreviations are used in this manuscript:

ABR	Auditory Brainstem Response
ALL	Acute Lymphoblastic Leukemia
AML	Acute Myeloid Leukemia
BOR	Brain–Lung–Thyroid
BPD	Bronchopulmonary Dysplasia
CT	Computed Tomography
HFNC	High-Flow Nasal Cannula
HGMD	Human Gene Mutation Database
ILD	Interstitial Lung Disease
MAS	Meconium Aspiration Syndrome
MRI	Magnetic Resonance Imaging
NICU	Neonatal Intensive Care Unit
PCR	Polymerase Chain Reaction
PDA	Patent Ductus Arteriosus
PTD	Partial Tandem Duplications
WES	Whole-Exome Sequencing
WSS	Wiedemann–Steiner Syndrome
